# Impact of decision to delivery time of fetal mortality in canine caesarean section in a referral population

**DOI:** 10.1002/vms3.163

**Published:** 2019-03-06

**Authors:** Lauren A. Proctor‐Brown, Soon Hon Cheong, Mariana Diel de Amorim

**Affiliations:** ^1^ Department of Clinical Sciences College of Veterinary Medicine Cornell University Ithaca New York USA

**Keywords:** Anesthesia time, canine, cesarean section, dystocia, perinatal death, surgery time

## Abstract

In human medicine, there is a recommended decision to delivery interval (DDI), which allows for the optimization of protocols and systematic review of hospital success. In veterinary medicine, no such guideline has been established or investigated. The purpose of this study was to investigate the relationship between the interval from the decision to perform a caesarean section and the delivery of the neonates and fetal mortality at the time of surgery. One hundred and fifty canine caesarean sections were evaluated retrospectively. Caesarean cases were dichotomized to those that had at least one perinatal death and cases where all puppies survived. Factors that increased the likelihood of at least one perinatal death at caesarean section were: cases that presented as an emergency caesarean section, the dam presenting with a fetus in the vaginal canal, the dam not having a history of previous caesareans sections, and being multiparous. Even though there was no association of DDI with likelihood of having perinatal death, timing related factors that increased the likelihood of having at least one perinatal death at caesarean section were: cases where total anaesthesia time was longer than 2 h; time from induction to start of surgery was longer than 45 min and surgical time longer than 75 min. In conclusion, time is a factor in the success of canine caesarean sections and further research is needed to better define the optimal decision to delivery time in canine caesarean sections in order to optimize fetal survival and hospital protocol success rates.

## Introduction

The estimated prevalence of dystocia in the canine, which requires veterinary attention may vary between 3.7% and 5% (Bergström *et al*. 2006; O'Neill *et al*. 2017) and 60–80% of dystocia cases require surgical intervention in the form of caesarean section (Darvelid & Linde‐Foresberg 1994; Bergström *et al*. 2006; Traas 2008a; Smith 2012). Uterine inertia is the most common maternal cause of dystocia in older bitches (Smith 2007; Linde‐Forsberg 2009), others include fetal to pelvic size mismatch. Fetal causes of dystocia include; malpresentation, malformation and fetal oversize. In a large population study, there was an increase in dystocia incidence for brachycephalic and mesocephalic breeds (Darvelid & Linde‐Foresberg 1994; Bergström *et al*. 2006).

It is a common practice to perform elective caesarean sections for certain brachycephalic breeds. In elective caesarean sections, there is a 5% mortality rate that significantly increases when litter sizes are over nine neonates (Batista *et al*. 2014). Indications to perform an emergent caesarean section include: fetal distress defined as fetal heart rate on ultrasounds <180 beats per minutes (bpm), primary and secondary uterine inertia and obstructive dystocia (Smith 2012). In canine populations, perinatal mortality or the sum of stillborn and neonatal deaths is relatively high; being reported up to 40% of the cases (Traas 2008a; Tønnessen *et al*. 2012). Additionally, canine parturition is a long process and fetal asphyxia during parturition is thought to be the most common cause of perinatal death (Moon *et al*. 1998; Traas 2008b). Prolonged dystocia >4.5–6 h or prolonged labour >10 h had an increased incidence of fetal mortality at delivery (Traas 2008a; Münnich & Küchenmeister 2009).

The American College of Obstetricians and Gynaecologists recommends an interval of no >30‐min from the decision to perform caesarean section to the delivery of the fetus in human obstetrical care facilities (Sayegh *et al*. 2004; Huissoud *et al*. 2010). This 30‐min guideline does not, however, correlate with outcome, rather an interval of 75‐min is correlated with poorer survival in terms of fetal Apgar scores and survival (Thomas *et al*. 2004). It is known that a human fetus will undergo cardiac arrest after 185 min of *in utero* distress, but may develop neurological deficits after 60 min of fetal distress (Lavery 1999). The presence of an on‐site anaesthesia team contributed to a faster decision to delivery interval (DDI) in human studies (Thomas *et al*. 2004; Huissoud *et al*. 2010). Furthermore, general anaesthesia decreased the overall outcome of caesarean sections for both mother and babies (Thomas *et al*. 2004; Huissoud *et al*. 2010).

In veterinary medicine, while a rapid intervention in cases of dystocia is recommended (Smith 2012), the decision to delivery time for canine patients has not previously been investigated. One study investigated the induction to delivery time and found it not to affect puppy vigour (Moon‐Massat 2002).

The purpose of this study was to investigate the relationship between the interval from decision to perform a caesarean section and the successful delivery of all canine neonates. In addition, we aimed to investigate the differences in survival between emergent and elective caesarean sections and described the morbidities and mortalities in our population of caesarean sections.

## Material and methods

### Data collection and case definition

The medical records of dogs having undergone caesarean section were identified from June of 2007 to March of 2017. Case data collected included: (1) bitch signalment, (2) whelping information and (3) time. Signalment data evaluated were: size (toy, small, medium, large and giant based on American Kennel Club breed standards), brachycephalic breed (Evans & de Lahunta 2013) (Yes or No), age (in years), parity, and previous caesarean sections (Yes or No). Whelping information evaluated were: emergent or elective procedure, the presence of a puppy in the vaginal canal (Yes or No), if the bitch had delivered at least one puppy vaginally before caesarean section (Yes or No), fetal compromise (defined as at least one fetus with consistent heart rate of <180 bpm), if medical management was attempted (Yes or No), anaesthetic complications (Yes or No), and maternal complications (Yes or No). Anaesthetic complications included: hypotension <60 mm Hg which required treatment with drugs such as dobutamine, glycopyrrolate, plasma, vasopressin and/or hetastarch; metabolic acidosis; hypothermia (<95°F); cardiac arrest; abnormal electrocardiogram; severe bradycardia; and/or hypoxemia that required oxygen administration). Maternal complications include those syndromes or negative effects of having undergone caesarean section; including but not limited to; incisional complications, infection, wound dehiscence, as well as maternal complications under anaesthesia that required treatment postoperatively. Timing data evaluated were: time from arrival to induction of anaesthesia, time from induction until the start of surgery, the duration of surgery until recovery from anaesthesia, and total time under general anaesthesia. The dependent variable was a dichotomous variable for puppy outcome defined as at least one perinatal death delivered at caesarean section (Yes or No). Perinatal death was defined as a puppy born dead or a puppy that died in the hospital shortly after resuscitation attempt. A positive puppy outcome was defined as a puppy that was discharged alive from the hospital. In cases where all neonates were known to be deceased at presentation were excluded from further analysis.

### Statistical analysis

Individual variables were first tested for association with the dependent variable, which was if at least one perinatal death was identified at caesarean section using logistic regression with JMP Pro version 13 (SAS Institute, Cary, NC, USA). Covariates with association with the dependent variable at *P* < 0.20 were offered to the model build. These were: breed, parity, previous caesarean section, emergency or elective, the presence of a puppy in the birth canal and if at least one puppy had been delivered vaginally before caesarean section. Two‐way interactions were also tested and offered to model build if *P* < 0.05.

Time variable was first evaluated for association with the dependent variable and the effects of covariates as a continuous variable using a linear regression ANOVA and the optimal threshold for each time variable was determined using Receiver Operator Characteristics (ROC) curve in JMP Pro version 13. The optimal time threshold was rounded to the nearest 15‐min time increment. The dichotomized time variable with an association with the dependent variable (*P* < 0.20) was offered to build the final logistic regression ANOVA model using backward‐stepwise procedure and retained if *P* < 0.05.

## Results

### Descriptive statistics

Summary statistics of the study variables are provided to describe the study population (Table 1). One hundred ninety‐two cases were identified, 42 were excluded due to insufficient information available. In one case, the dam did not survive and was excluded. Fifty‐three breeds were represented; the most common were the English Bulldog (*n* = 15) American Bulldog (*n* =14) and Labrador Retriever (*n* = 15). Fifty‐nine (40%) cases were of a brachycephalic breed, 23 (39%) of those cases were elective caesareans. Fifty‐eight (38.7%) cases were known to be multiparous and of those 25 (43%) had a previous caesarean section, 69 (46%) cases were known to be primiparous and the remaining 23 cases (15.3%) had an unknown reproductive history.

**Table 1:** Demographic data of combined, emergent and elective c‐section

	All	Emergency	Elective
Age (years)^a^	3 (0.92–12)	3 (0.92–10)	3 (1.5–12)
Parity			
Primiparous	69 (46%)	44 (54%)	25 (57%)
Multiparous	58 (38.7%)	39 (46%)	19 (43%)
Unknown	23 (15.3%)		
Previous C‐section	25 (42%, *n* = 60)	13 (32%, *n* = 41)	12 (63%, *n* = 19)
Brachycephalic	59 (40%)	36 (61%)	23 (39%)
Breed category			
Toy	12 (8%)	12 (11.5%)	0
Small	26 (17.3%)	17 (16.3%)	9 (20%)
Medium	38 (25.3%)	18 (17.3%)	20 (43%)
Large	50 (33.3%)	42 (40.3%)	8 (17%)
Giant	24 (16%)	15 (14.5%)	9 (20%)
Stage on presentation			
Not in labour	34 (23%)	3 (4%)	31 (67%)
Stage 1	45 (30%)	31 (30%)	14 (30%)
Stage 2	71 (47%)	70 (67%)	1 (2%)
Fetal distress (HR <180 bpm)	53 (64%, *n* = 83)	45 (73%, *n* = 62)	8 (38%, *n* = 21)
Neonates delivered vaginally	46 (31%)	45 (43%)	1 (2%)
Neonates delivered alive (#)^a^, ^b^	3.5 (0–13)	3 (0–11)	4.5 (1–13)
Anaesthetic complications	100 (67%)	68 (65%)	32 (70%)
Hypotension	93 (62%)	63 (50%)	30 (65%)
Maternal complications	28 (19%)	21 (20%)	7 (15%)
Anaesthesia time (min)^a^	105 (45–225)	111 (45–200)	91.5 (60–225)
Arrival to induction (min)^a^	132 (27–1358)	115 (27–1206)	189.5 (56–1358)
Induction to surgery (min)^a^	35 (10–70)	35 (10–70)	35.5 (10–65)
Surgery time (min)^a^	71.6 (15–70)	73.8 (15–150)	66.5 (20–170)
Neonatal death at caesarean	52 (35%)	45 (43%)	7 (15%)
Total number of cases	150	104 (69%)	46 (31%)

a Data displayed in median (range).

b Number of neonates that were delivered alive per bitch.

### Clinical presentation and management

The majority of emergent cases presented in labour, besides three which were not in stage 2 labour; one presented for vomiting and progressed to labour while being monitored, the other two were not in labour but proceeded to caesarean when signs of fetal distress were seen on ultrasound. Of the elective cases only one presented in stage 2 labour; an English Bulldog being monitored for elective caesarean but presented immediately after delivering one puppy vaginally at home. Of the 70 cases presenting in stage 2 labour for emergent caesarean section, 45 (60%) had already delivered at least one puppy vaginally and 38 (54%) presented with a puppy in the vaginal canal. Twelve cases (8%) were managed medically before proceeding to surgery, seven cases received oxytocin and were monitored while five cases were only monitored and stabilized. In 16 emergent cases (15%) vaginal assisted delivery was attempted, but only in six cases (38%) was a puppy delivered successfully. Abdominal ultrasound to assess for fetal distress was performed in 83 cases (55%) and of those 53 (64%) had a lowest fetal heart rate below 180 bpm (Table 1).

### Clinical outcome

Of the 150 cases, 65 (43%) had at least one perinatal death, and in one case there was maternal death. In the case of the maternal death, three stillborn puppies were delivered at surgery and the dam became septic secondary to a ruptured uterus and suffered sudden cardiac arrest the day following surgery. In 19 (13%) cases at least one neonate was stillborn delivered vaginally with or without assistance prior to surgery. At surgery 53 (37%) additional cases where at least one neonate was delivered deceased and 8 (5%) cases no neonates were successfully delivered alive.

Anaesthetic complications were seen in 100 (66%) cases and were represented most commonly by hypotension (93 cases). Other anaesthetic complications seen included; two cases with severe acidosis requiring bicarbonate administration, hypothermia, cardiac arrest, AV block, ventricular escape beats, atrial fibrillation, bradycardia, hypoxemia on extubation requiring intervention and gastric dilatation without torsion requiring oro‐gastric intubation. Maternal complications were seen in 28 (19%) of cases and included persistent anaemia, haemorrhage, septic peritonitis, uterine rupture, atrial fibrillation, uterine infection, wound dehiscence, seroma at the incisional site, regurgitation and secondary aspiration pneumonia, euthanasia 2 days post surgery due to septic peritonitis, cardiac arrest under anaesthesia with recovery after cardiopulmonary resuscitation, death reported at home 2 days post surgery.

### Neonatal outcome

Bivariate analysis for the association between having at least one perinatal death and covariates are shown in Table 2. Two‐way interactions were not significant. Time from arrival to anaesthetic induction was not associated with puppy outcome individually and not retained in the final model. The ROC analysis showed the optimal threshold for anaesthesia time to be 117 min with a sensitivity (Se) and specificity (Sp) of 53.9% and 71.4% area under the curve (AUC) = 66.9%, *P *= 0.0011 and was rounded to 120 min. Bitches that had anaesthetic time ≥120 min had 6.67 odds ratio (95% CI 2.64–18.25, *P* < 0.0001) of at least one perinatal dead puppy being delivered by caesarean section. Results of the full model for total anaesthesia time are summarized in Table 3.

**Table 2:** Bivariate analysis of the complete and emergent data

	Complete data			Emergent		
	*P*‐values	OR	95% CI	*P*‐values	OR	95% CI
Emergency	0.0005					
No	Ref	Ref	Ref			
Yes	0.0005	4.25	1.83–11.17			
Brachycephalic	0.87			0.5543		
No	Ref	Ref	Ref	Ref	Ref	Ref
Yes	0.87	0.95	0.47–1.88	0.55	1.28	0.56–2.89
Breed category	0.19			0.2824		
Toy	Ref	Ref	Ref	Ref	Ref	Ref
Small	0.03	0.22	0.05–0.91	0.17	0.35	0.07–1.57
Medium	0.02	0.2	0.05–0.78	0.23	0.4	0.08–1.76
Large	0.04	0.24	0.06–0.86	0.039	0.25	0.06–0.93
X large	0.09	0.3	0.06–1.24	0.48	0.57	0.11–2.7
Age	0.4			0.6735		
Parity	0.13			0.2572		
Previous C section	0.017			0.5325		
No	Ref	Ref	Ref	Ref	Ref	Ref
Yes	0.06	0.36	0.01–1.03	0.3869	0.578	0.15–1.96
NA	0.08	2.23	0.91–5.58	0.59	1.3	0.49–3.43
Neonates vaginal	0.13			0.8327		
Vaginally assisted	0.8			0.6107		
Medical management	0.6			0.905		
Stage	0.0048			0.1268		
Stage 1	Ref	Ref	Ref	Ref	Ref	Ref
Stage 2	0.0103	2.84	1.27–6.69	0.0634	2.31	0.96–5.94
None	0.68	0.8	0.26–2.32	0.2	4.89	0.42–113.23
Puppy in canal	0.0236			0.2938		
No	Ref	Ref	Ref	Ref	Ref	Ref
Yes	0.024	2.39	1.13–5.13	0.29	1.54	0.69–3.46
Fetal distress	0.05			0.0065		
No	Ref	Ref	Ref	Ref	Ref	Ref
Yes	0.53	1.38	0.51–3.7	0.915	1.06	0.36–3.17
NA	0.02	2.57	1.16–5.92	0.007	3.79	1.43–10.69
Arrival to induction (continuous)	0.45			0.17		
Arrival to induction ≥90 min	0.1356			0.36		
<90 min	Ref	Ref	Ref	Ref	Ref	Ref
≥90 min	0.14	0.56	0.26–1.2	0.36	0.67	0.29–1.56
Arrival to surgery	0.41			0.15		
Anaesthesia time (continuous)	<0.0011			0.0039		
Anaesthesia time ≥120 min	0.0017			0.0043		
<120 min	Ref	Ref	Ref	Ref	Ref	Ref
≥120 min	0.0017	3.46	1.59–7.69	0.0043	3.7	1.5–9.72
Induction to surgery (continuous)	0.18			0.29		
Induction to surgery ≥45	0.0137			0.0294		
<45 min	Ref	Ref	Ref	Ref	Ref	Ref
≥45 min	0.0137	2.91	1.25–6.93	0.0294	3.02	1.12–8.79
Surgery to recovery (continuous)	0.0008			0.0024		
Surgery to recovery ≥75	0.0028			0.0039		
<75 min	Ref	Ref	Ref	Ref	Ref	Ref
≥75 min	0.0028	2.86	1.43–5.79	0.0039	3.21	1.45–7.34

**Table 3:** Final model for the association between the time from anaesthesia induction to recovery with at least one stillborn puppy delivered by caesarean section

	Odds ratio	95% CI	*P*
All			
Anaesthesia time ≥120 min	6.67	2.64–18.25	<0.0001
Parity – primiparous	0.232574	0.08–0.64	0.0047
Previous caesarean section – yes	0.18	0.04–0.65	0.0081
Puppy in canal – yes	2.62	1.13–6.20	0.0248
Emergency only			
Anaesthesia time ≥120 min	3.70	1.50–9.72	0.0043

When total anaesthesia time was divided into the time from anaesthesia induction to the start of surgery ≥45 min (ROC 48 min, Se 28.9%, Sp = 87.7%, AUC = 55.2%; OR = 2.84, 95% CI 1.16–7.19, *P* = 0.0228), and the time from the start of surgery to anaesthesia recovery ≥75 min (ROC 76 min, Se = 55.8%, Sp = 69.4%, AUC = 65.8%; OR = 2.34, 95% CI 1.13–4.90, *P* = 0.0026), both were associated with increased odds of having at least one perinatal death at caesarean section. Cases presenting as an emergency were more likely to have neonatal mortality (OR = 3.73, 95% CI 1.54–10.15, *P* = 0.0029). The full model for the separated total anaesthesia time is summarized in Table 4.

**Table 4:** Final model for the association between the time from induction to surgery and the time from surgery to recovery with at least one stillborn puppy delivered by caesarean section

	Odds ratio	95% CI	*P*
All			
Time from induction to surgery ≥45 min	2.84	1.16–7.19	0.0228
Time from surgery to recovery ≥75 min	2.34	1.13–4.90	0.0026
Emergency – yes	3.73	1.54–10.15	0.0029
Emergency only			
Time from induction to surgery ≥45 min	3.13	1.11–9.55	0.031
Time from surgery to recovery ≥75 min	3.29	1.45–7.70	0.0041

### Emergency cases only

Bivariate analysis for the association between having at least one perinatal death at caesarean section and covariates in emergency cases is summarized in Table 2. The interval between arrival and anaesthesia induction was not associated with having at least one perinatal death at caesarean section (*P* = 0.36). The final model for anaesthesia time ≥120 min retained only this variable (OR = 3.70, 95% CI 1.50–9.72, *P* = 0.0043).

When the total anaesthesia time was divided into induction to surgery and surgery to recovery, both time from induction to surgery ≥45 min (OR = 3.13, 95% CI 1.11–9.55, *P* = 0.031) and time from surgery to recovery ≥75 min (OR = 3.29, 95% CI 1.45–7.70, *P* = 0.0041) were significantly associated with having at least one perinatal death at caesarean section.

## Discussion

This is the first study to examine the relationship of the decision‐to‐delivery interval in canine patients and fetal survival at caesarean section and documents that an increase in anaesthesia time is associated with an increase in fetal mortality. When examining emergent cases alone, longer surgery time was associated with increased odds of fetal death. In our population, time from arrival to the hospital to induction of anaesthesia was not associated with increased foetal death. Time is an important factor to maximize fetal survival in canine feti but further research is needed to determine the optimal decision‐to‐delivery interval guideline in canine caesarean sections.

In our population, the neonatal and maternal mortality at caesarean section is in line with previously published studies in which mortality rates at caesarean section range from 1% to 33% (Moon *et al*. 2000; Batista *et al*. 2014). As the cost of breeding and whelping well‐bred puppies is increasing the loss of even one puppy from a litter can have a large economic impact of responsible breeders who are relying on veterinarians to achieve the best possible outcome for the dam and litter (Smith 2007). As the demand to decrease neonatal mortality has increased, optimizing caesarean section protocol to improve neonatal survival is a necessity.

Defining the DDI for canine patients in this study was difficult due to the implications of a retrospective review of medical records. Therefore, the overall‐time from arrival to induction and induction to start of surgery was used as a proxy for our canine patients. Our results demonstrated that the time from arrival to induction and induction to start of surgery was significantly longer than those recommended in humans. This is likely due to key differences between canine and human parturition that impact the length of the necessary DDI. Furthermore, elective caesarean sections may have a longer DDI without necessarily affecting puppy survival. Parturition in litter‐bearing species is a long process and stage 2 labour can last 3 to 12 h averaging one puppy per hour (Smith 2012) and even longer when the litter is large or the dam is primiparous. Further research on fetal distress and DDI could help define the optimal DDI for canine patients. Key factors that impacted DDI in human parturition include the availability of trained personnel, type of anaesthesia and the awareness of staff. It has been shown that improving staff awareness of protocols can significantly shorten the DDI (Lurie *et al*. 2004).

The association between increased duration of total anaesthesia and increased neonatal mortality could be confounded by other factors but also lends weight to the urgency with which a caesarean section should be performed. Factors such as surgical complications, large litter size, and anaesthetic complications could impact the duration of anaesthesia and worsen the surgical outcomes. This study did not attempt to examine anaesthetic protocols as they are formulated on an individual case basis by the dedicated anaesthesia clinicians at this hospital. As such, there is not an enforced anaesthetic protocol for caesarean section, though alpha‐2 agonists, ketamine, and thiobarbiturates are generally avoided (Moon *et al*. 2000; Traas 2008a; Smith 2012). There is a decrease in fetal Apgar scores when using propofol for induction as compared with alfaxalone; however no long‐term difference in the outcomes was identified (Doebeli *et al*. 2013), and both induction agents were used in this study. In Human medicine, standardizing the anaesthetic process appeared to have a significant impact in optimizing DDI (Huissoud *et al*. 2010) and could be considered when developing a protocol in canine caesarean management.

We determined a few risk factors that could assist clinicians in identifying dystocia cases that are at higher risk of neonatal fatality and should be considered to shorten the DDI. Dams presenting with a fetus lodged in the canal were significantly more likely to have neonatal mortality at caesarean section. There was no association with fetal distress, defined as a fetal heart rate <180 bpm on ultrasound, but this is likely due to the low numbers of cases for which this was recorded. These parameters together could aid clinicians in determining when to focus on shortening the DDI in order to optimize fetal survival at surgery. It has been suggested in human studies that different DDI guidelines for different levels of urgency could be more clinically useful than one strict guideline (Sayegh *et al*. 2004). In our population, there was a higher likelihood of decrease in fetal survival in emergent cases. Bitches that had previous caesarean sections were more likely to have no fetal mortality from caesarean section compared with bitches that did not have previous caesarean sections. Conversely, multiparous bitches were more likely to have fetal mortality compared with primiparous bitches. This finding is in contrast to another study that found primiparous bitches to have an increased likelihood of stillbirth (Münnich & Küchenmeister 2009).

The limitations of this study include an inability to prove causation due to its retrospective nature and lack of a control population. In addition, we were limited to using the start of surgery as a proxy for time of delivery as that was most commonly recorded in the medical records; and due to the fact that just over half of the patients had an ultrasound for fetal viability prior to surgery, some of the perinatal death may have occurred prior to surgery and so the data should be interpreted carefully. Furthermore, the population in this study was biased towards brachycephalic elective caesarean sections, which are known to have an increased risk of neonatal mortality (Bergström *et al*. 2006). This study aimed to investigate if there was a relationship between DDI and fetal survival at the time of caesarean section; the statistical analysis supports a correlation between increased duration of anaesthetic and surgical time with increased fetal mortality, but there was no association of increased DDI and neonatal mortality. Due to the retrospective nature of this research, the optimal DDI timing was unable to be evaluated. Future clinical studies could investigate causation and optimal DDI to maximize fetal survival or document methods in which to improve DDI in canine patients.

## Conclusion

While further research is needed to define an optimal decision‐to‐delivery interval recommendation for canine dystocias, total anaesthesia time should likely not exceed longer than 2 h. In particular, time from induction to start of the surgery should not exceed 45 min, because longer durations are associated with increased fetal mortality rates in our population. Furthermore, in urgent dystocias, bitches that had no previous caesarean sections and with obstructive dystocia (puppy in the canal) every attempt should be made to shorten the DDI. Hospital protocols improving staff awareness have shown to improve the DDI in human medicine and could be extrapolated to veterinary medicine to improve canine DDI.

## Source of funding

Funding for publication costs was provided from the start‐up research fund from Dr Diel de Amorim.

## Conflicts of interest

None of the authors have any conflict of interest to declare.

## Ethics statement

Due to the retrospective nature of this study, an Animal Care Protocol was not required.

## Contributions

Dr Proctor‐Brown has conducted the review of the medical record, collected the data and wrote the first draft of the manuscript. Drs Cheong has performed the statistical analysis. Diel de Amorim has mentored the writing of the first daft. All authors have contributed in the study design and have approved the final manuscript.
